# The Psychosocial Outcome of Conduct and Oppositional Defiant Disorder in Children With Attention Deficit Hyperactivity Disorder

**DOI:** 10.7759/cureus.9521

**Published:** 2020-08-02

**Authors:** Noha Eskander

**Affiliations:** 1 Psychiatry, California Institute of Behavioral Neurosciences & Psychology, Fairfield, USA

**Keywords:** psychology, conduct disorder, attention-deficit/ hyperactivity disorder, oppositional defiant disorder, psychiatry, child and adolescent psychiatry, psychosocial, neurology, attention deficit hyperactivity disorder (adhd), psychiatry and mental health

## Abstract

Attention deficit hyperactivity disorder (ADHD) is one of the most common mental disorders diagnosed in children below the age of 12 years. It is characterized by hyperactivity, inattention, and impulsive behavior. ADHD affects the social, academic, and psychological aspects of children and adolescents. Children with ADHD struggle with school tasks and performance. They have lower grades than their peers and have difficulties interacting with their friends.

Oppositional defiant disorder (ODD) is a mental disorder characterized by disruptive behavior, a pattern of angry and irritable mood, argumentative, and vindictive behavior. Children with ODD struggle with forming friendships and have problems at school. Conduct disorder (CD) is divided into the childhood onset and the adolescent onset types. The childhood onset is associated with poor outcomes in adulthood, an increase in criminal behavior, violence, and progression to antisocial behavior. Children with CD are at increased risk for substance use disorders (SUD) and antisocial personality disorder.

The current literature review is aiming to provide an overview of the psychosocial impact of comorbid ODD and CD in children with ADHD. The results of this study review showed the comorbidity of ODD and CD is very strong. ODD is a strong predictor of CD in boys. The presence of comorbid ODD and ADHD in children is a significant predictor of adolescent onset CD. The comorbidity of ADHD with ODD and CD worsens symptom severity and is associated with high psychosocial dysfunction. Children with ADHD and comorbid ODD and CD have difficulties with school, friends, and trouble with the police.

## Introduction and background

Attention deficit hyperactivity disorder (ADHD) is a neurodevelopmental disorder diagnosed in children below the age of 12 years. It is characterized by difficulty paying attention, excessive activity, disruptive behavior, and impulsivity. The prevalence of ADHD among children and adolescents is 7.2% [1]. It is four times more common in males compared to females in a community sample [2]. Approximately half of the children diagnosed with ADHD continue to have symptoms as adults [3]. Children with ADHD suffer from learning disabilities and a decrease in school performance. They also struggle with psychosocial functioning in the family. The disorder is associated with familial low socioeconomic disadvantages, such as low income, single parenthood, and lower level of education [4]. ADHD is frequently associated with comorbid disorders like anxiety disorders, conduct disorder (CD), oppositional defiant disorder (ODD), and substance abuse disorder [5].

CD is a mental and behavioral disorder in children. Children with CD suffer from disruptive behavior and have problems with following rules. Children with this disorder have repetitive and persistent patterns of aggression, destruction, and deceitfulness. CD is twice more common in boys compared to girls. The prevalence of CD in the United States is 6% to 16% in males and 2% to 9% in females. Symptoms appear earlier in boys at a median age of 11 years compared to girls with a median age of 15 years. Children with CD have educational difficulties; they often struggle with psychosocial functioning and isolation. Other psychiatric comorbidities are common with CD, such as depression, ADHD, substance abuse, and ODD [6].

ODD is a disruptive behavioral disorder in children; it consists of a pattern of angry and irritable mood, argumentative, and vindictive behavior [7]. The lifetime prevalence of ODD is 10.2%. Males are more affected than females with 11.2% and 9.2%, respectively. A diagnosis of ODD made before the age of eight years indicates a poor prognosis [8].

CD and ODD frequently coexist together. ODD is believed to be a risk factor to CD [7]. ODD co-occurs in half of the children with ADHD, while CD co-occurs in 20% of children with ADHD [9].

The objective of this literature review is to explore the impact of comorbid disorders like CD and ODD on children and adolescents diagnosed with ADHD. We are looking to increase our understanding of how children with these comorbid disorders function socially, psychologically, and academically comparable to children with ADHD only. Our study review aims to find out why these disorders commonly occur together and what common risk factors that might coexist. We have reviewed past research studies that discussed ADHD, CD, and ODD as separate disorders and as comorbid disorders.

## Review

Methods and results

Data were searched on PubMed using regular keywords: "Attention deficit hyperactivity disorder (ADHD)", "Conduct disorder (CD)", "Oppositional defiant disorder (ODD)". Table 1 shows the search results of the regular keywords "Attention deficit hyperactivity disorder (ADHD)", "Conduct disorder (CD)", "Oppositional defiant disorder (ODD)".

**Table 1:** PubMed keywords search results

Keywords	Database	Date	Number of results
Attention deficit hyperactivity disorder (ADHD)	PubMed	7/11/2020	37,126
Oppositional defiant disorder (ODD)	PubMed	7/11/2020	1,150
Conduct disorder (CD)	PubMed	7/11/2020	1,244

Results

The total number of keywords search results was 39,520. The following is a breakdown of the keywords searched and the volume of results: Attention deficit hyperactivity disorder (ADHD) keyword search results was 37,126· Conduct disorder (CD) keyword search results was 1,244· Oppositional defiant disorder (ODD) keyword search results was 1,150

Only research articles related to human studies that were published since 2002 in the English language were included in this study. All types of research articles were included except for books and documents. After the manual screening of each article, the relevant research studies for this literature review were selected. A total of 31 articles were selected for this study to determine the impact of comorbid CD and ODD in children with ADHD on psychosocial functioning and behavioral adjustment.

Discussion

Based on the study results, ADHD is associated with impairment in psychosocial functioning. Children with comorbid CD, ODD, and ADHD have significant learning and intellectual disabilities. Children with these disorders struggle psychologically, socially, and academically.

*Attention Deficit Hyperactivity Disorder* 

ADHD is divided into three subtypes: predominantly inattentive, predominantly hyperactive-impulsive, or combined. Children with ADHD struggle to perform academically in school. They typically have lower scores and grades compared to their peers and have higher rates of school dropouts. The poor academic performance is linked to a lack of attention rather than hyperactivity. Girls are more likely to struggle with attention problems compared to boys who struggle with hyperactivity and impulsivity. About 20% to 25% of children with ADHD have learning disabilities [10]. There is a strong correlation between reading disabilities and attentiveness. In a sample of children with ADHD and learning disabilities, 15% to 50% had reading disabilities, 24% to 60% had difficulties in math, and 60% had problems with spelling [11].

Children and adolescents with ADHD struggle socially and are often isolated from their peers. They have high conflict levels within their families. This behavior is possibly due to the difficulty in emotion regulation [12]. Children with attention deficits have difficulties in processing verbal and nonverbal languages, which affect social interactions. Adolescents with ADHD commonly miss social cues, and they fall behind adolescents’ complex communication and nuances. Their poor social skills are often mistakenly perceived as a lack of empathy. Those factors affect the self-esteem, confidence, and the emotional and mental being of adolescents with ADHD [13]. The executive function and the working memory are both impaired in ADHD that leads to the inability to learn new academic skills, store information, and move toward achieving goals [10]. Executive function impairment is present in 30% to 50% in children with ADHD [14]. ADHD neuroimaging studies showed deficits in the subcortical regions such as the basal ganglia and the insula. The studies also showed volume reductions in the hippocampus and the amygdala [15]. 

*Conduct Disorder* 

CD is divided into childhood onset and adolescent onset types. The onset of CD in childhood is associated with poor outcomes in adulthood, such as an increase in criminal behavior, violence, and progression to antisocial behavior. Children with CD are also at an increased risk for substance use disorders (SUD) and personality disorders such as antisocial personality disorder [16]. Neuroimaging studies in children with CD revealed a smaller size of the amygdala, the insula, the ventromedial prefrontal cortex, and the orbitofrontal cortex. There is evidence of a decrease in responses in these brain regions that are responsible for the social behavior in CD [17]. There is a decrease in the level of serotonin and cortisol and a reduction in the autonomic nervous system (ANS) functions in CD. These findings possibly explain the dysregulation in mood, impulse control, and the decrease in the signals of anxiety and fear in CD [18].

Oppositional Defiant Disorder

According to the Diagnostic and Statistical Manual of Mental Disorders, Fifth Edition (DSM-V), ODD consists of three dimensions: irritability and anger, vindictiveness, and argumentative behavior. Children with ODD struggle with forming friendships and have problems in school [19]. Comorbid disorders, such as anxiety disorders, SUD, and mood disorders, are commonly associated with ODD. The presence of comorbid disorders together with symptom onset of ODD below the age of eight years predicts high morbidity [8]. Unlike CD, ODD does not include aggression towards people, animals, or property destruction. Children with ODD have an overactive Behavioral Activation System (BAS), which is an index to sensitivity to reward, and underactive Behavioral Inhibition System (BIS), which is sensitive to signals of punishment [20].

Comorbidity of CD and ODD

CD and ODD are disruptive behavior disorders that commonly coexist with 96% in the clinical samples and 60% in the general population. ODD is a strong predictor of CD in boys but not in girls. Some studies found CD is associated with behavioral problems, while ODD is more commonly linked to emotional regulation issues. However, more recent evidence has indicated that ODD includes both emotional and behavioral problems. ODD was considered a mild form of CD that forms at an early developmental stage. The Diagnostic and Statistical Manual of Mental Disorders, Fourth Edition (DSM-IV) mentioned that all features of ODD are essentially present in CD, and diagnosis of CD essentially includes ODD [21].

Whether CD and ODD are completely separate disorders or expressions of one underlying disorder is a cause of scientific debates. Some argued that ODD is an exaggeration of common features like the rebellious behavior that happens normally in adolescents; therefore, it should be considered as a temperament dimension, not a separate categorical disorder. The repetitive pattern of behavior in which there is a violation of the basic rights of others is an important aspect that characterizes CD. Although both CD and ODD are more common in boys compared to girls, CD is significantly more common in boys than ODD. A longitudinal study (n=8,000) showed ODD develops at a median age of 2.5 years compared to CD that develops at a median age of 5.5 years. CD is associated with a high risk of SUD such as alcohol use disorder (AUD) and tobacco. The comorbidity of the two disorders increases the risk of SUD. Children with comorbid CD and ODD are at high risk to develop antisocial personality disorder, anxiety, and depression [21]. 

Comorbidity of ADHD, CD, and ODD

ADHD, CD, and ODD were under one heading in the DSM-IV: “Attention Deficit and Disruptive Disorders". In DSM-V, ADHD is considered a neurodevelopment disorder. CD and ODD are classified as disruptive, impulse-control, and conduct disorders.

ADHD and ODD: About 47% of children with ADHD have ODD [22]. Those children with comorbid ADHD and ODD display severe symptoms of both disorders more than those with ADHD or ODD alone. They have difficulty interacting with their peers and have high developmental deficits [23]. According to a cross-sectional study (n=64), children with ADHD and ODD have more internalized symptoms, such as higher rates of depression and anxiety, than those with ODD or ADHD alone. They have more problems in school with their teachers and with their mothers at home [24]. Children and adolescents with ADHD and ODD share common developmental risk factors like a positive family history of ADHD or ODD, maternal smoking during pregnancy, and the presence of family conflict. The risk of development of ODD is associated with family violence, a decrease in parental affection, and association with deviant peers [25].

ADHD and CD: About 30% to 50% of children with ADHD have comorbid CD. Some studies showed the presence of ADHD predicts antisocial behavior and is an indicator of the severity of the symptoms developed in CD. Children with ADHD and comorbid CD have higher rates of criminal behavior as adults, SUD, and school dropouts [26]. While ADHD and CD share some common risk factors like low family socioeconomic status and parental conflict, the risk of developing CD is associated with antisocial peers, high neighborhood crime, and cold parental attitude [25,27].

The presence of comorbid ODD and ADHD in children is a significant predictor of adolescent onset CD type, especially in females [28]. ADHD, ODD, and CD share similarities in risk factors, genetic components, and symptoms, which suggest the possibility of a common psychopathological spectrum. A common pattern exists in children with these comorbid disorders; the pattern usually starts with ADHD symptoms, progressing to ODD and then developing into CD later on. Family studies proved that ADHD, ODD, and CD can occur together in certain families, which suggests a common etiology. Genetic studies showed CD is linked to ADHD hyperactive-impulsive type with genetic hereditability of 37%. Similarly, ODD is linked to ADHD hyperactive-impulsive type with a genetic hereditability of 42%. Both ODD and CD have a lower genetic link with ADHD-inattentive and ADHD-combined types [29].

A cross-sectional study (n=1,029) found out that children with ADHD and intellectual disability are at significant risk to develop ODD and show more symptoms of comorbid CD [30]. ADHD is commonly associated with reading disability. Children with reading disabilities are as three times as likely to develop comorbid ADHD, CD, and ODD [11]. Table 2 summarizes some of the important studies used to describe the comorbidity in ADHD, ODD, and CD in children and adolescents.

**Table 2:** Summary of some of the studies used in reviewing the comorbidity of ADHD, ODD, and CD ADHD: attention deficit hyperactivity disorder, ODD: oppositional defiant disorder, CD: conduct disorder

Author name	Year of publication	Study design	Sample size (if applicable)	Conclusions
Van Lier et al. [28]	2007	Observational study (cross-sectional)	2,076	ADHD commonly occurs with CD. The presence of ODD predicts this occurrence in children and adolescents. There is a gender difference in the occurrence of CD.
Ghosh et al. [29]	2012	Case report	3	ADHD, ODD, and CD share common developmental pathways and symptoms; therefore, these findings suggest a common psychopathological spectrum in these disorders.
Ahuja et al. [30]	2013	Observational study (cross-sectional)	1,029	Children with ADHD and intellectual disability have high rates for comorbid CD and ODD.
Padhy et al. [11]	2015	Review	Not applicable	Reading disability commonly occurs with psychological comorbidities, such as internalizing disorders (depression and suicide) and externalizing disorders (ADHD, ODD, and CD).

**Figure 1:**
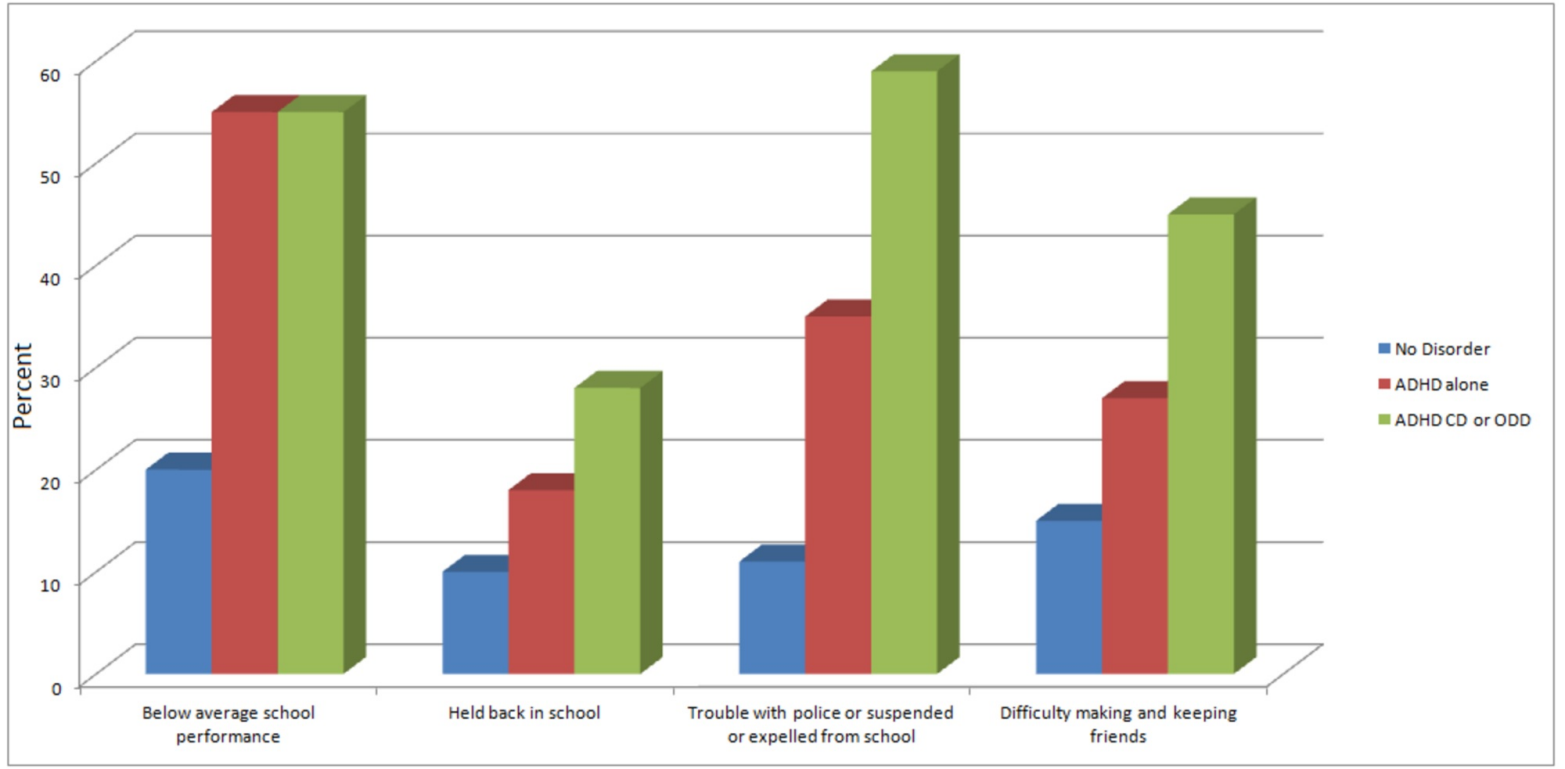
School performance and social function in children with no disorder compared to children with ADHD and children with ADHD, ODD and CD ADHD, attention deficit hyperactivity disorder, ODD: oppositional defiant disorder, CD: conduct disorder

The comorbidity of ADHD with ODD and CD worsens symptom severity and is associated with high psychosocial dysfunction (Figure 1). Figure 1 shows children with ADHD struggle in school and have difficulty making friends. Children with ADHD and ODD and/or CD are the most group that have difficulties with school, friends, and trouble with the police.

Study Limitation

Our study is based on reviewing research studies in the last 18 years, so possibly we missed other important contributions from studies before this. We did not perform a systematic review in our study and no quality assessment of the selected research studies was done.

## Conclusions

ADHD is a common neurodevelopmental disorder in children. Comorbid disorders, such as CD, ODD, anxiety disorders, and learning disabilities, are frequently associated with ADHD. ODD and CD are disruptive disorders that usually coexist together. SUD and antisocial personality disorder are comorbid disorders with ODD and CD. Children and adolescents with ADHD, CD, and ODD are at an increased risk to develop other problems like anxiety disorders, SUD, and mood disorders. Those children struggle to function among their peers, family, and school. Learning and intellectual disabilities are also common. ADHD, CD, and ODD share common risk factors, genetics, and symptoms. 

Children presented with ADHD should be carefully screened for the presence of other comorbid disorders like CD and ODD. Properly analyzing and addressing the common psychosocial risk factors is a crucial step in the prevention of these comorbid disorders. Creating educational programs and support groups to ease the burden of the comorbid disorders on the families of the affected children is necessary. Creating effective networking between families, teachers, and health care providers are essential. Further research studies are required to address the genetic and neurobiological aspects of these comorbid disorders.
